# The activation of CD14, TLR4, and TLR2 by mmLDL induces IL-1β, IL-6, and IL-10 secretion in human monocytes and macrophages

**DOI:** 10.1186/1476-511X-9-117

**Published:** 2010-10-14

**Authors:** Luis Chávez-Sánchez, Karina Chávez-Rueda, Maria Victoria Legorreta-Haquet, Edgar Zenteno, Yadira Ledesma-Soto, Eduardo Montoya-Díaz, Emiliano Tesoro-Cruz, Alejandra Madrid-Miller, Francisco Blanco-Favela

**Affiliations:** 1 Unidad de Investigación Médica en Inmunología, Hospital de Pediatría, Centro Médico Nacional Siglo XXI, IMSS, México D. F., México; 2 Unidad de Cuidados Intensivos Cardiovasculares, Hospital de Cardiología, Centro Médico Nacional Siglo XXI, IMSS, México D. F., México; 3 Departamento de Bioquímica, Facultad de Medicina, UNAM, México, D. F., México

## Abstract

Atherosclerosis is considered a chronic inflammatory disease in which monocytes and macrophages are critical. These cells express CD14, toll-like receptor (TLR) 2, and TLR4 on their surfaces, are activated by minimally modified low-density lipoprotein (mmLDL) and are capable of secreting pro-inflammatory cytokines. The aim of this research was thus to demonstrate that the activation of CD14, TLR2, and TLR4 by mmLDL induces the secretion of cytokines.

**Methods:**

Human monocytes and macrophages were incubated with monoclonal antibodies specific for CD14, TLR4, and TLR2 prior to stimulation with mmLDL. Cytokine secretion was then compared to that observed upon mmLDL stimulation in untreated cells.

**Results:**

Stimulation with mmLDL induced the secretion of pro-inflammatory cytokines. Blocking CD14 in monocytes inhibited secretion of interleukin (IL)-1β (72%), IL-6 (58%) and IL-10 (63%), and blocking TLR4 inhibited secretion of IL-1β by 67%, IL-6 by 63% and IL-10 by 60%. Blocking both receptors inhibited secretion of IL-1β by 73%, IL-6 by 69% and IL-10 by 63%. Furthermore, blocking TLR2 inhibited secretion of IL-1β by 65%, IL-6 by 62% and IL-10 by 75%. In macrophages, we found similar results: blocking CD14 inhibited secretion of IL-1β by 59%, IL-6 by 52% and IL-10 by 65%; blocking TLR4 inhibited secretion of IL-1β by 53%, IL-6 by 63% and IL-10 by 61%; and blocking both receptors inhibited secretion of IL-1β by 69%, IL-6 by 67% and IL-10 by 65%. Blocking TLR2 in macrophages inhibited secretion of IL-1β by 57%, IL-6 by 40% and IL-10 by 72%.

**Conclusion:**

Our study demonstrates that CD14, TLR4, and TLR2 participate in the immune response against mmLDL by inducing the production of pro-inflammatory cytokines in both monocytes and macrophages. These findings suggest that the activation of these receptors by mmLDL contributes to the inflammatory process of atherosclerosis.

## Introduction

Several studies have shown that pro-inflammatory cytokines, such as tumor necrosis factor (TNF)-α, interleukin (IL)-1β, and IL-6, play an important role in the development of atherosclerosis [1]. Monocytes and macrophages are innate immune cells that are central to the inflammatory response in the atherosclerotic plaque. These cells are the main producers of pro-inflammatory cytokines [2,3] during the response to exogenous antigens that are involved in atherosclerosis, such as *Chlamydia pneumoniae *[4], or to endogenous antigens such as oxidized low-density lipoprotein (oxLDL), which has been shown to play a role in the development of atherosclerotic plaques [5,6]. Furthermore, oxLDL is considered a pro-atherogenic molecule [7] that is capable of inducing the secretion of TNF-α [8].

Monocytes and macrophages express CD14 and toll-like receptors (TLRs) on the cell surface [9]. CD14 and TLRs are pattern recognition receptors capable of activating multiple genes that encode pro-inflammatory cytokines such as IL-1β and IL-6, adhesion molecules such as cellular-1 vascular adhesion molecule and intracellular-1 adhesion molecule, and co-stimulatory molecules such as CD80 in response to pathogens or molecular patterns associated with pathogens [10]. Some studies have demonstrated the participation of the TLRs in the development of the atherosclerotic plaque [11,12], and previous evidence suggests a potential role for oxidized modified LDL as an endogenous antigen capable of triggering and maintaining the inflammatory process in the atherosclerotic plaque [5-7]. Previous studies have also demonstrated that minimally modified low-density lipoprotein (mmLDL) induces TLR4-dependent secretion of MIP-2 and TLR4-independent, MyD88-independent secretion of TNF-α in macrophages [13]. In contrast, we and other authors have reported that the synthesis of TNF-α is dependent on TLR4 [14,15]. Furthermore, we demonstrated that TLR2 also participates in the synthesis of TNF-α in response to mmLDL [14].

The regulation of the activation of TLRs includes several mechanisms such as the production of anti-inflammatory cytokines, mainly IL-10 [16]. Or well by the TLRs homologs such as RP105 protein, that interacts directly with the TLR4 signaling complex, resulting in the negative regulation of TLR4 [17]. The production of these negative regulators, assures proper regulation of the pro- and anti-inflammatory balance [16].

In this study, we aimed to analyze the participation of CD14, TLR4, and TLR2 in the production of the pro-inflammatory cytokines IL-1β and IL-6 and the anti-inflammatory cytokine IL-10 in response to mmLDL. We found that blocking these receptors inhibited the production of IL-1β, IL-6, and IL-10. These results provide new perspectives on the role of oxidized modified LDL in the inflammation associated with atherosclerosis.

## Materials and methods

Informed consent was obtained from seven healthy, normolipidemic 20- to 30-year-old male volunteers without cardiovascular risk factors or clinically apparent atherosclerotic disease. The study was approved by the Human Ethics and Medical Research Committee of the Instituto Mexicano del Seguro Social and conducted according to the guidelines of the Declaration of Helsinki.

### LDL isolation and modification

Native human LDL (nLDL) (density = 1.019-1.063 g/ml) was isolated from plasma by ultracentrifugation. The density of the plasma was adjusted to 1.2 g/ml by adding solid potassium bromide (KBr) (J.T. Baker, Phillipsburg, NJ). Density gradients were formed in 6-ml polycarbonate centrifuge tubes by overlaying 2 ml plasma solution with 3 ml 0.5 mM NaCl (endotoxin-free), pH = 7.4, d = 1.006 g/ml. The tubes were ultracentrifuged in a Sorvall Discovery M150 SE ultracentrifuge equipped with an S80AT3 fixed angle rotor at 41,500 × g for 4 hours at 4°C. After ultracentrifugation, very low-density lipoprotein was removed, 3.0 ml of the lower layer was transferred to another tube, 2 ml KBr-NaCl (d = 1.18 g/ml) was added and the samples were gently mixed. The tubes were ultracentrifuged at 41,500 × g for 4 hours at 4°C, and the LDL fraction was removed. The LDL fraction was dialyzed against 0.5 mM NaCl, pH = 7.4, for 24 hours at 4°C to remove EDTA. LDL was oxidized using CuSO_4 _(J.T. Baker). To produce mmLDL, 300 μg/ml LDL was incubated with 10 μmol/L CuSO_4 _(J.T. Baker) for 1 hour at 37°C. The mmLDL was then extensively dialyzed against 0.5 mM NaCl, 0.5 mM EDTA, pH = 7.4, for 24 hours at 4°C. Oxidative modification of LDL was assessed by measuring thiobarbituric acid-reactive substances as previously described [18]. This assay showed that samples contained between 0.8 and 2.8 nmol malondialdehyde per milligram of protein. The optimal concentrations of mmLDL for activation of human monocytes and macrophages were 50 μg/ml and 70 μg/ml, respectively (data not shown). All nLDL and mmLDL preparations used in these experiments were tested for bacterial lipopolysaccharide (LPS) contamination using a Limulus Amoebocyte Lysate kit (BioWhittaker, Walkersville, MD) according to the manufacturer's instructions.

### Monocyte isolation

Peripheral blood mononuclear cells (PBMCs) were obtained from buffy coats by density centrifugation using Lymphoprep (Axis-Shield, Oslo, Norway). The buffy coats were mixed with an equal volume of phosphate buffered saline (PBS), pH = 7.4, layered over 3 ml of Lymphoprep and centrifuged at 700 × g for 30 minutes. The recovered PBMCs were washed three times with PBS, pH = 7.4. The monocytes were then isolated from PBMCs by negative selection. The PBMCs were incubated with a cocktail of biotin-conjugated antibodies against CD3, CD7, CD16, CD19, CD56, CD123 and glycophorin A and magnetic microbeads coupled to an anti-hapten monoclonal antibody and depleted using a magnetic column (Monocyte Isolation Kit II, Miltenyi Biotec, Bergisch Gladbach, Germany). Purified cells were stained for CD14, and the purity of monocytes was determined by flow cytometry to be >98%.

### Differentiation of U937 cells to macrophages

To produce macrophages, U937 cells (ATCC) were adjusted to 2 × 10^6^/ml in RPMI 1640 medium (Invitrogen, Carlsbad, CA) containing 10% fetal calf serum and phorbol myristate acetate (PMA) (Sigma-Aldrich, St. Louis, MO) at a final concentration of 2.5 ng/ml and incubated for 24 hours at 37°C. The adherent cells were washed three times with PBS, pH = 7.4, to remove PMA and incubated in culture medium containing 10% fetal calf serum without PMA for an additional 24 hours at 37°C before being used in experiments. The maturation of U937 cells was evaluated by measuring their CD14 expression by flow cytometry.

### Ability of anti-CD14, -TLR4, and -TLR2 antibodies to inhibit monocytes and macrophages activation

Monocytes and macrophages were cultured using RPMI 1640 medium in 96-well plates at a concentration of 2 × 10^5^/well. For blocking experiments, the monocytes and macrophages were preincubated for 1 hour at 37°C with 10 μg/ml anti-CD14 (clone MEM18, BD Biosciences, San Jose, CA), anti-TLR4 (clone HTA125, Santa Cruz Biotechnology, Santa Cruz, CA), or a combination of both antibodies for 1 hour at 37°C. After washing both cells three times with PBS, pH = 7.4, monocytes and macrophages were stimulated with 100 ng/ml LPS (Sigma-Aldrich, St. Louis, MO) (positive control) for 24 hours at 37°C. Alternatively, monocytes and macrophages were treated with 10 μg/ml anti-TLR2 (clone TL2.1, eBioscience, San Diego, CA) for 1 hour at 37°C and stimulated with 20 ng/ml PamCys (synthetic lipopeptide Pam3Cys-Ser-Lys4) (Alexis Biochemicals, San Diego, CA) (positive control). As an additional control, both cells were treated with 10 μg/ml irrelevant antibodies before stimulation with TLRs ligands. The culture supernatants were collected after 24 hours of incubation at 37°C. Secreted IL-1β, IL-6, and IL-10 levels were determined by Cytometric Bead Array (CBA) kit (BD Biosciences) according to the manufacturer's instructions (Additional files 1 and 2).

### Blocking CD14, TLR4 and TLR2

Human monocytes (2 × 10^5^/ml) and U937 cells (2 × 10^5^/ml) were treated with 10 μg/ml human anti-CD14 antibody (clone MEM18, BD Biosciences), human anti-TLR4 antibody (clone HTA125, Santa Cruz Biotechnology), or a combination of both antibodies for 1 hour at 37°C. After washing the cells three times with PBS, pH = 7.4, monocytes were stimulated with 50 μg/ml mmLDL, and macrophages were stimulated with 70 μg/ml mmLDL for 24 hours at 37°C. Alternatively, human monocytes (2 × 10^5^/ml) and U937 cells (2 × 10^5^/ml) were incubated with 10 μg/ml anti-TLR2 (clone TL2.1, eBioscience) for 1 hour at 37°C, washed three times with PBS, pH = 7.4, and stimulated with 50 μg/ml mmLDL (monocytes) or 70 μg/ml mmLDL (macrophages) for 24 hours at 37°C. As a positive control for TLR4 and TLR2 activation, monocytes and macrophages were stimulated with 100 ng/ml LPS (Sigma-Aldrich) and 20 ng/ml PamCys (Alexis Biochemicals). As an antibody control, cells were treated with irrelevant IgG isotype control antibodies before stimulation with TLRs ligands or mmLDL. As a negative control, monocytes and U937 cells were incubated only in culture medium or with control antibodies in the absence of TLRs agonist or mmLDL stimulation. All supernatants were frozen at -70°C prior to determination of cytokines secretion.

### Cytokine analysis

Cytokines were measured in culture supernatants of monocytes or macrophages treated with 50 μg/ml or 70 μg/ml mmLDL, respectively, or in cells treated with only culture medium or with control antibodies (10 μg/ml) using a CBA kit (BD Biosciences) and flow cytometry analysis, according to the manufacturer's instructions. Briefly, the cytokine standards were diluted (5 to 5000 pg/ml). The standard dilutions and test samples were added to the appropriate sample tubes (50 μl) and mixed with 50 μl each of antibody-PE detector and antibody-bead reagent (50 μl). The mixture (150 μl) was incubated for 3 hours in the dark at room temperature and washed. The test samples were acquired using a FACS Calibur flow cytometer (BD Biosciences).

### Statistical analysis

The Mann-Whitney test was used to evaluate the statistical significance of differences between experimental groups. Samples with *P *< 0.05 were considered significantly different. The data shown in all figures are expressed as the mean ± SEM of values from independent experiments.

## Results

### Ability of anti-CD14, -TLR4, and -TLR2 antibodies to inhibit monocytes and macrophages activation in responses to LPS and Pamcys

As a positive control, we stimulated TLR4 with LPS and TLR2 with PamCys in monocytes and macrophages. We found that pre-treatment with anti-CD14, anti-TLR4, or a combination of both antibodies inhibited secretion of IL-1β (Additional file 1: Figure S1A and S1C), IL-6 (Additional file 1: Figure S1B and S1D) and IL-10 (Additional file 2: Figure S2A and S2B) in monocytes and macrophages, respectively, stimulated with LPS. Similarly, blocking TLR2 prior to PamCys stimulation inhibited secretion of IL-1β (Additional file 1: Figure S1A and S1C), IL-6 (Additional file 1: Figure S1B and S1D) and IL-10 (Additional file 2: Figure S2A and S2B) in monocytes and macrophages, respectively.

### Inhibition of IL-1β production in monocytes and macrophages by blocking CD14 and TLR4

**Figure 1:**
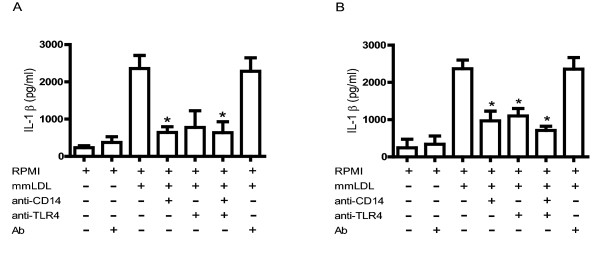
**Role of CD14 and TLR4 in secretion of IL-1β in response to mmLDL**. Human monocytes (1A) and macrophages (1B) were treated with anti-CD14, anti-TLR4, or both antibodies (10 μg/ml) for 1 hour before incubation with mmLDL (50 μg/ml and 70 μg/ml, respectively). Monocytes and macrophages were incubated with irrelevant antibody (10 μg/ml) in the presence or absence of mmLDL (50 μg/ml and 70 μg/ml, respectively). The concentration of IL-1β in culture supernatants was determined by CBA. *p < 0.005.

Stimulation with mmLDL also induced considerable production of IL-1β compared with the negative control (cells treated only with culture medium). Blocking CD14 in monocytes (Figure 1A) and macrophages (Figure 1B) prior to mmLDL stimulus inhibited IL-1β production by 72% and 59%, respectively. Meanwhile, blocking TLR4 in monocytes and macrophages inhibited IL-1β production by 67% and 53%, respectively. When we blocked both receptors in monocytes and macrophages, we found a 73% and 69% reduction, respectively, in IL-1β secretion.

### Blocking CD14 and TLR4 inhibits IL-6 secretion in monocytes and macrophages

**Figure 2:**
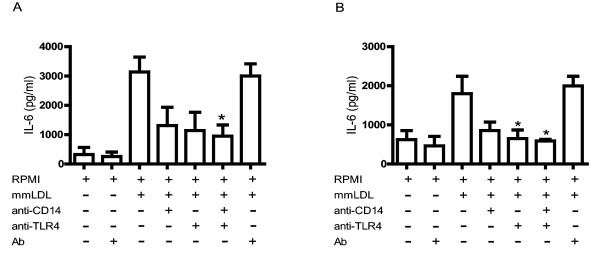
**Role of CD14 and TLR4 in secretion of IL-6 in response to mmLDL**. Human monocytes (2A) and macrophages (2B) were treated with anti-CD14, anti-TLR4, or both antibodies (10 μg/ml) for 1 hour before incubation with mmLDL (50 μg/ml and 70 μg/ml, respectively). Monocytes and macrophages were incubated with irrelevant antibody (10 μg/ml) in the presence or absence of mmLDL (50 μg/ml and 70 μg/ml, respectively). The concentration of IL-6 in culture supernatants was determined by CBA. *p < 0.005.

Stimulation of monocytes and macrophages with mmLDL caused an increase in the secretion of IL-6 at levels higher than those observed in cells cultured only with culture medium. Blocking CD14 in monocytes (Figure 2A) and macrophages (Figure 2B) caused a 58% and 52% reduction in IL-6 secretion, respectively. When both cell types were treated with anti-TLR4 prior to stimulation with mmLDL, IL-6 secretion was inhibited by 63% and 63%, respectively. Blocking both receptors inhibited IL-6 secretion by 69% and 67%, respectively, after a 24-hour culture period.

### Blocking TLR2 inhibits IL-1β and IL-6 secretion in monocytes and macrophages

**Figure 3:**
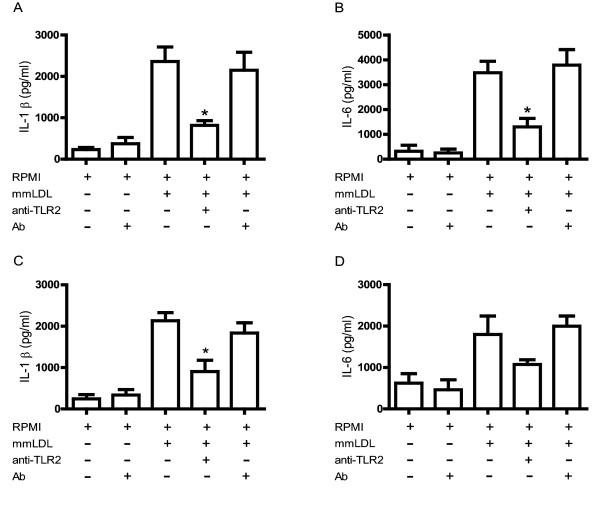
**Role of TLR2 in secretion of IL-1β and IL-6 in response to mmLDL**. Human monocytes and macrophages were treated with anti-TLR2 (10 μg/ml) for 1 hour before stimulation with mmLDL (50 μg/ml and 70 μg/ml, respectively). Monocytes and macrophages were incubated with irrelevant antibody (10 μg/ml) in the presence or absence of mmLDL (50 μg/ml and 70 μg/ml, respectively). The concentrations of IL-1β and IL-6 in culture supernatants of monocytes (3A and 3B) and macrophages (3C and 3D) were determined by CBA, respectively. *p < 0.005.

Stimulation with mmLDL induced increases in IL-1β and IL-6 secretion in monocytes and macrophages when compared to cells cultured only with culture medium. Preincubation with anti-TLR2 induced a 65% and 57% reduction in IL-1β secretion in monocytes (Figure 3A) and macrophages (Figure 3C), respectively. Similarly, the secretion of IL-6 in response to mmLDL was reduced by 62% and 40% in monocytes (Figure 3B) and macrophages (Figure 3D), respectively.

### The roles of CD14, TLR4, and TLR2 in the production of IL-10 in response to mmLDL

**Figure 4:**
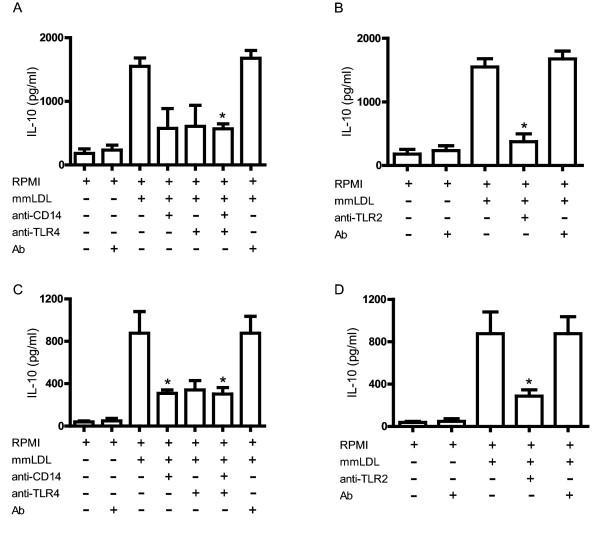
**Role of CD14, TLR4, and TLR2 in secretion of IL-10 in response to mmLDL**. Human monocytes (4A) and macrophages (4C) were treated with anti-CD14, anti-TLR4, or both antibodies (10 μg/ml) for 1 hour before incubation with mmLDL (50 μg/ml and 70 μg/ml, respectively). Monocytes (4B) and macrophages (4D) were treated with anti-TLR2 (10 μg/ml) for 1 hour before stimulation with mmLDL (50 μg/ml and 70 μg/ml, respectively). Monocytes and macrophages were incubated with irrelevant antibody (10 μg/ml) in the presence or absence of mmLDL (50 μg/ml and 70 μg/ml, respectively). The concentration of IL-10 in culture supernatants was determined by CBA. *p < 0.005.

Stimulation of monocytes and macrophages with mmLDL induced secretion of IL-10 at levels higher than those observed in cells cultured only with culture medium. Blocking CD14 in monocytes (Figure 4A) and macrophages (Figure 4C) inhibited IL-10 secretion by 63% and 65%, respectively, blocking TLR4 inhibited IL-10 secretion by 60% and 61%, respectively, and blocking both receptors inhibited IL-10 secretion by 63% and 65%, respectively. Similarly, preincubation of monocytes (Figure 4B) and macrophages (Figure 4D) with anti-TLR2 antibody inhibited mmLDL-mediated IL-10 secretion by 75% and 72%, respectively.

## Discussion

Atherosclerosis is currently considered a chronic inflammatory disease in which monocytes and macrophages are critical [1-3]. The possible etiologies of this disease include infections with *Chlamydia pneumoniae *or responses to endogenous antigens such as LDL [5]. Several studies have shown that the oxidized modifications of LDL influence the development of atherosclerotic lesions through the inflammatory response [5-7]. We previously demonstrated that mmLDL can activate CD14, TLR4, and TLR2, inducing TNF-α [14]. Here we clearly demonstrate that mmLDL elicits the production of IL-1β, IL-6, and IL-10 through CD14, TLR4, and TLR2 in human monocytes and macrophages.

CD14 has been proposed as the first host pattern recognition receptor involved in the recognition of most bacterial components [19,20] and facilitates the expression of inflammatory molecules via activation of the TLRs [21]. Our results show that monocytes and macrophages are activated by a very early form of oxidized LDL via CD14, which is a necessary co-receptor for mmLDL-mediated secretion of IL-1β and IL-6. These findings are consistent with our previous study, in which we showed that blocking CD14 in monocytes and macrophages stimulated with mmLDL resulted in significant inhibition of TNF-α production [14]. These results are supported by the finding that blocking CD14 in monocytes stimulated with extensively oxidized LDL resulted in significant inhibition of IL-6 and IL-1β production [22], suggesting that activation through the CD14 pathway plays a role in inflammation in response to mmLDL.

TLRs are an essential part of the innate immune system [9,10]. Several reports indicate that TLRs can recognize endogenous ligands such as heat shock protein (HSP) 60 and hyaluronic acid, thereby inducing the expression of TNF-α, IL-6, and other pro-inflammatory cytokines [10,23,24]. Our results show that mmLDL induced IL-1β and IL-6 production through TLR4. This finding is supported by previous studies in which we demonstrated that mmLDL induced TNF-α through TLR4 [14]. Both of these results are supported by the fact that blocking TLR4 inhibits TNF-α production in cells stimulated with advanced glycation end-product of low-density-lipoprotein [15].

The role of the CD14/TLR4 pathway in the immune response to endogenous antigens such as mmLDL is not well understood. Here we examined whether the activation of CD14/TLR4 induced the secretion of IL-1β and IL-6 in cells stimulated with mmLDL. Our results showed that mmLDL elicited the production of IL-1β and IL-6 and that this response was inhibited when CD14 and TLR4 were blocked. These findings are similar to previous reports that extensively oxidized LDL induces the secretion of IL-6 and IL-1β in monocytes [22] and that blocking CD14 and TLR4 before stimulation with mmLDL affects the secretion of TNF-α [14], suggesting that the activation of the CD14/TLR4 pathway by mmLDL triggers an immune-mediated response driving synthesis of pro-inflammatory cytokines.

We have demonstrated that TLR2 induces TNF-α in response to mmLDL [14]. Here we show that the secretion of IL-6 and IL-1β by monocytes and macrophages is TLR2-dependent, suggesting that TLR2 participates in the secretion of pro-inflammatory cytokines in response to mmLDL. This function is similar to the TLR2 response to other endogenous antigens, such as HSP60, which also induced the secretion of TNF-α [25].

Our results indicate that the activation of CD14, TLR4, and TLR2 on monocytes and macrophages by a very early form of oxidized LDL induces the secretion of pro-inflammatory cytokines such as IL-1β and IL-6, which may contribute to or exacerbate inflammatory responses during atherosclerosis. Both of these cytokines activate endothelial cells, inducing the expression of chemokines such as monocyte chemoattractant protein-1, which recruits monocytes to the lesion area, and the upregulation of adhesion molecules, which facilitate adhesion of leukocytes to endothelial cells [26,27]. Moreover, the exogenous administration of IL-6 exacerbates the atherosclerotic lesion [28], and deficiency in IL-1β reduces atherosclerotic lesion size [29].

Previous evidence demonstrates that stimulation with extensively oxidized LDL induces the secretion of IL-10 [30], and early forms of oxidized LDL induce an increase in IL-10 mRNA expression in macrophages [31]. Moreover, monocytes and macrophages secrete IL-10 upon activation through TLR2 or TLR4 [32]. In the present study, we found that mmLDL induced CD14-, TLR4-, and TLR2-dependent production of IL-10. Furthermore, we demonstrated that blocking TLR2 affected IL-10 secretion by monocytes and macrophages more than blocking CD14, TLR4, or both CD14 and TLR4, suggesting that mmLDL-mediated production of IL-10 may regulate cellular activation. During the initial activation phase, macrophages secrete pro-inflammatory cytokines [33], and the second phase of macrophage activation involves the delayed and gradual production of IL-10 [34].

## Conclusions

Our results establish a potential pathogenic mechanism by which activation of CD14, TLR4, and TLR2 by mmLDL initiates or exacerbate the pro-inflammatory state in atherosclerotic disease.

## List of Abbreviations

TLR: toll-like receptor; mmLDL: minimally modified low-density lipoprotein; IL: interleukin; TNF: tumor necrosis factor; oxLDL: oxidized low-density lipoprotein; nLDL: native LDL; LPS: lipopolysaccharide; PBMCs: peripheral blood mononuclear cells; PMA: phorbol myristate acetate; PamCys: synthetic lipopeptide Pam3Cys-Ser-Lys4; CBA: cytometric bead array.

## Competing interests

The authors declare no conflicts of interest.

## Authors' contributions

CS and BF conceived the idea and designed the experiments. CS performed the experiments. CR, LH, and ZE participated in the experiments related to flow cytometer. LS and MD participated in the obtained of LDL. TC and MM contributed in the performed the statistical analysis. CS, BF, and ZE and drafted the manuscript and interpretation of the data. All authors read and approved the final manuscript.

## Supplementary Material

Additional file 1 Inhibition of IL-1β and IL-6 production in monocytes and macrophages by blocking CD14, TLR4, and TLR2. Click here for file

Additional file 2 Inhibition of IL-10 production in monocytes and macrophages by blocking CD14, TLR4, and TLR2. Click here for file

## References

[B1] Hanson GK Robertson AK Söderberg-Nauclér C Inflammation and atherosclerosis Annu Rev Pathol 2006 1 297 329 10.1146/annurev.pathol.1.110304.100100 18039117

[B2] Woollard KJ Geissmann F Monocytes in atherosclerosis: subsets and functions Nat Rev Cardiol 2010 7 77 86 10.1038/nrcardio.2009.228 20065951 PMC2813241

[B3] Tabas I Macrophage deaht and defective inflammation resolution in atherosclerosis Nat Rev Immunol 2010 10 36 46 10.1038/nri2675 19960040 PMC2854623

[B4] Netea MG Kullberg BJ Jacobs LE Verver-Janseb TJ van der Ven-Jongekrijg J Galama JM Stalenhoef AF Dinarello CA Van der Meer JW *Chamydia pneumoniae *stimulates IFN-gamma synthesis through Myd88-dependent, TLR2 and TLR4 independent induction of IL-18 release J Immunol 2004 15 1477 1482 10.4049/jimmunol.173.2.1477 15240744

[B5] Ross R The pathogenesis of atherosclerosis: a perspective for the 1990s Nature 1993 362 801 809 10.1038/362801a0 8479518

[B6] Libby P Inflammation in atherosclerosis Nature 2002 420 868 874 10.1038/nature01323 12490960

[B7] Virella G Atchley D Koskinen S Zheng D Lopes-Virella MF Proatherogenic and proinflammatory properties of immune complexes prepared with purified human oxLDL antibodies and human oxLDL Clin Immunol 2002 105 81 92 10.1006/clim.2002.5269 12483997

[B8] Jovinge S Ares Mp Kallin B Nilsson J Human monocytes/macrophages release TNF-alpha in response to oxLDL Arterioscler Thromb Vasc Biol 1996 16 1573 1579 8977464 10.1161/01.atv.16.12.1573

[B9] Uematsu S Akira S Toll-like receptors and innate immunity J Mol Med 2006 9 712 725 10.1007/s00109-006-0084-y 16924467

[B10] Takeda Akira S Toll-like receptors in innate immunity Int Immunol 2005 17 1 14 10.1093/intimm/dxh186 15585605

[B11] Mullick AE Tobias PS Curtiss LK Modulation of atherosclerosis in mice by Toll-like receptor 2 J Clin Invest 2005 115 3149 3156 10.1172/JCI25482 16211093 PMC1242192

[B12] Michelsen KS Wong MH Shah PK Zhang W Yano J Doherty TM Akira S Rajavashisth TB Arditi M Lack of Toll-like receptor 4 or mice deficient in apolipoprotein E Proc Natl Acad Sci USA 2004 101 10685 10690 10.1073/pnas.0403249101 15249654 PMC489994

[B13] Miller YI Viriyakosol S Worrall DS Boullier A Butler S Witztum JL Toll-like receptor 4-dependent and -independent cytokine secretion induced by minimally oxidized low-density lipoprotein in macrophages Arterioscler Thromb Vasc Biol 2005 25 1213 1219 10.1161/01.ATV.0000159891.73193.31 15718493

[B14] Chávez-Sánchez L Madrid-Miller A Chávez-Rueda K Legorreta-Haquet MV Tesoro-Cruz E Blanco-Favela F The activation of TLR2 and TLR4 by minimally modified LDL in human macrophages and monocytes triggers the inflammatory response Human Immunol 2010 8 737 744 10.1016/j.humimm.2010.05.005 20472010

[B15] Hodgkinson CP Laxton RC Patel K Ye S Advanced glycation end product of low density lipoprotein activates the Toll like 4 receptor pathway implications for diabetic atherosclerosis Arterioscler Thromb Vasc Biol 2008 28 2275 2281 10.1161/ATVBAHA.108.175992 18818414

[B16] Liew FY Xu D Brint EK O'Neill LA Negative regulation of toll-like receptor-mediated immune responses Nat Rev Immunol 2005 6 446 458 10.1038/nri1630 15928677

[B17] Iwami KI Matsuguchi T Masuda A Kikuchi T Musikacharoen T Yoshikai Y Cutting edge: naturally occurring soluble form of mouse Toll-like receptor 4 inhibits lipopolysaccharide signaling J Immunol 2000 165 6682 6686 11120784 10.4049/jimmunol.165.12.6682

[B18] Pentikainen MO Lindsted KA Kovanen PT Inhibition of the oxidative modification of LDL by nitecapone Arterioscler Thromb Vasc Biol 1995 15 740 747 7773727 10.1161/01.atv.15.6.740

[B19] Dziarski R Ulmer AJ Gupta D Interactions of CD14 with components of gram-positive bacteria Chem Immunol 2000 74 83 107 full_text 10608083 10.1159/000058761

[B20] Pugin J Heumann ID Tomasz A Kravchenko VV Akamatsu Y Nishjima M Glause MP Tobias PS Ulevitch RJ CD14 is a pattern recognition receptor Immunity 1994 1 509 516 10.1016/1074-7613(94)90093-0 7534618

[B21] Segura M Vadeboncoeur N Gottschalk M CD14-dependent and-independent cytokine and chemokine production by human THP-1 monocytes stimulated by *Strptococcus suis *capsular type 2 Clin Exp Immunol 2002 127 243 254 10.1046/j.1365-2249.2002.01768.x 11876746 PMC1906344

[B22] Pasini AF Anselmi M Garbin U Franchi E Stranieri C Nava MC Boccioletti V Vassanelli C Cominacini L Enhance levels of oxidized low-density lipoprotein prime monocytes to cytokine overproduction via upregulation of CD14 and Toll-like receptor 4 in unstable angina Arterioscler Thromb Vasc Biol 2007 27 1991 1997 10.1161/ATVBAHA.107.142695 17600225

[B23] Kol A Lichtman AH Finberg RW Libby P Kurt-Jones EA Cutting edge: heat shock protein (HSP) 60 activates the innate immune response: CD14 is an essential receptor for HSP60 activation of mononuclear cells J Immunol 2000 164 13 17 10604986 10.4049/jimmunol.164.1.13

[B24] Beg AA Endogenous ligands of Toll-like receptors: implications for regulating inflammatory and immune responses Trends Immunol 2002 23 509 512 10.1016/S1471-4906(02)02317-7 12401394

[B25] Iwahashi M Yamamura M Aita T Okamoto A Ueno A Ogawa N Akashi S Miyake K Godowski PJ Makino H Expression of Toll-like receptor 2 on CD16+ blood monocytes and synovial tissue macrophages in rheumatoid arthritis Arthritis Rheum 2004 50 1457 1467 10.1002/art.20219 15146415

[B26] Sims JE Smith DE The IL-1 family regulators of immunity Nat Rev Immunol 2010 10 89 102 10.1038/nri2691 20081871

[B27] Gabay C Interleukin 6 and chronic inflammation Arthritis Res Ther 2006 8 suppl 2 S3 10.1186/ar1917 16899107 PMC3226076

[B28] Huber SA Sakkien P Conze D Hardin N Tracy R Interleukin-6 exacerbates early atherosclerosis in mice Arterioscler Thromb Vasc Biol 1999 19 2364 2367 10521365 10.1161/01.atv.19.10.2364

[B29] Kirii H Niwa T Yamada Y Wada H Saito K Iwakura Y Asano M Moriwaki H Seishima M Lack of interleukin-1 beta decreases the severity of atherosclerosis in ApoE-deficient mice Arterioscler Thromb Vasc Biol 2003 23 656 660 10.1161/01.ATV.0000064374.15232.C3 12615675

[B30] Fei GZ Huang YH Swedendorg J Frostegård J Oxidised LDL modulates immune-activation by an IL-12 dependent mechanism Atherosclerosis 2003 169 77 85 10.1016/S0021-9150(03)00146-1 12860253

[B31] Bae YS Lee JH Choi SH Kim S Almazan F Witztum JL Miller YI Macrophages Generate Reactive Oxygen Species in Response to Minimally Oxidized Low-Density Lipoprotein: toll-like receptor 4- and spleen tyrosine kinase-dependent activation of NADPH oxidase 2 Circ Res 2009 104 210 218 10.1161/CIRCRESAHA.108.181040 19096031 PMC2720065

[B32] Chi H Barry SP Roth RJ Wu JJ Jones EA Bennett AM Flavell RA Dynamic regulation of pro- and anti inflammatory cytokines by MAPK phosphatase 1 (MKP-1) in innate immune responses Proc Natl Acad Sci USA 2006 104 2274 2279 10.1073/pnas.0510965103 PMC1413743 16461893

[B33] Dong C Davis RJ Flavell RA MAP kinases in the immune response Annu Rev Immunol 2002 20 55 72 10.1146/annurev.immunol.20.091301.131133 11861597

[B34] de Waal Malefyt R Abrams J Bennett B Figdor CG de Vries JE Interleukin 10 (IL-10) inhibits cytokine synthesis by human monocytes: an autoregulatory role of IL-10 produced by monocytes J Exp Med 1991 174 1209 1220 10.1084/jem.174.5.1209 1940799 PMC2119001

