# Supplementary material for: The activation of CD14, TLR4, and TLR2 by mmLDL induces IL-1β, IL-6, and IL-10 secretion in human monocytes and macrophages
Source: Lipids Health Dis. 2010 Oct 14;9:117. doi: 10.1186/1476-511X-9-117 (PMC2964726; doi:10.1186/1476-511X-9-117)
Supplement: Additional file 1 — Inhibition of IL-1β and IL-6 production in monocytes and macrophages by blocking CD14, TLR4, and TLR2. [file 1476-511X-9-117-S1.DOC]

**Additional file 1**

**Figure S1. Inhibition of IL-1β and IL-6 production in monocytes and macrophages by blocking CD14, TLR4, and TLR2**.

Human monocytes and macrophages were treated with anti-CD14, anti-TLR4, or both antibodies (10 μg/ml) for 1 hour before incubation with LPS (100 ng/ml). Alternatively, monocytes and macrophages were treated with 10 µg/ml anti-TLR2 for 1 hour at 37ºC and stimulated with 20 ng/ml PamCys. Both cells were incubated with irrelevant antibody (10 μg/ml) in the presence or absence of TLR ligands. Culture supernatants were collected after 24 hours of incubation at 37°C. The concentrations of IL-1β (1A and 1C) and IL-6 (1B and 2D) in the culture supernatants of monocytes and macrophages were determined by CBA, respectively. The values plotted in white bar are associated to the Y axis on the left. The values plotted on the gray bar are associated to the Y axis on the right. **p*<0.005.
